# 2DDB – a bioinformatics solution for analysis of quantitative proteomics data

**DOI:** 10.1186/1471-2105-7-158

**Published:** 2006-03-20

**Authors:** Lars Malmström, György Marko-Varga, Gunilla Westergren-Thorsson, Thomas Laurell, Johan Malmström

**Affiliations:** 1 Department of Electrical Measurements, LTH, P.O Box 118, SE-221 00, Lund, Sweden; 2 Department of Analytical Chemistry, Lund University, SE-221 87, Lund, Sweden; 3 Department of Cell and Molecular Biology, C13, BMC, University of Lund, SE-221 84, Lund, Sweden; 4 Institute for Molecular Systems Biology, ETH Hönggerberg, HPT E 53, Wolfgang Pauli-Str. 16, CH-8093 Zürich, Switzerland

## Abstract

**Background:**

We present 2DDB, a bioinformatics solution for storage, integration and analysis of quantitative proteomics data. As the data complexity and the rate with which it is produced increases in the proteomics field, the need for flexible analysis software increases.

**Results:**

2DDB is based on a core data model describing fundamentals such as experiment description and identified proteins. The extended data models are built on top of the core data model to capture more specific aspects of the data. A number of public databases and bioinformatical tools have been integrated giving the user access to large amounts of relevant data. A statistical and graphical package, R, is used for statistical and graphical analysis. The current implementation handles quantitative data from 2D gel electrophoresis and multidimensional liquid chromatography/mass spectrometry experiments.

**Conclusion:**

The software has successfully been employed in a number of projects ranging from quantitative liquid-chromatography-mass spectrometry based analysis of transforming growth factor-beta stimulated fi-broblasts to 2D gel electrophoresis/mass spectrometry analysis of biopsies from human cervix. The software is available for download at SourceForge.

## Background

A typical proteomics experiment starts with separation of the biological material of interest. Popular separation technologies include two-dimensional gel electrophoresis (2DE) and liquid chromatography (LC). After separation, the proteins are commonly identified by mass spectrometry (MS) which identifies proteins by measuring the weight of protein fragments (for example peptides from a tryptic digestion) and subsequently search these fragment weights against sequence databases. This search is carried out by software such as SEQUEST [1] or MASCOT [2]. The usage of sequence databases for protein identification forces the user to choose between quality or quantity. Highly curated databases such as Swiss-Prot [3] (the curated protein sequence database of UniProt) ensure high-quality protein identifications and makes subsequent analysis less difficult. A protein or peptide cannot be identified unless the protein or peptide sequence is present in the searched database, and hence the relative small size of Swiss-Prot (about 172000 protein sequences as of the spring of 2005; release 46.2) limits the number of proteins that can be identified for a given sample. Large databases such as the NCBI's non-redundant protein sequence database (NR) with about 2.3 million unique sequences and is composed of a large selection of other databases such as GenBank [4-6], Swiss-Prot [3], EMBL [7] and DDBJ [8]. The quality of annotations is on average of lower quality than in Swiss-Prot because of a higher degree of automation. The subsequent analysis of the data becomes more difficult but as the size of the database increases the chances of identifying proteins increase. Software has also been developed to further enhance the quality of the data by applying advanced statistics to the resulting protein and peptide identifications [9,10]. The resulting data consists of thousands of MS spectra, from various fractions of the sample, searched against one or more sequence databases using one or more software packages. There is a need to store this complex data and there is a need to find information about the identified proteins. This information can range from physical properties such as molecular weight and pI to what biological pathways this protein is a part of or what protein family it belongs to. Information can be extracted from on-line databases, some mentioned above or produced by bioinformatical tools.

The amount of data is at times large, but the nature of the data poses bigger challenges. The data acquisition is sequential meaning that sample preparation comes before sample separation, which in turn comes before identification. Each step can be performed by a variety of techniques and all steps are not performed in each experiment. Since the data from each step is different the software integrating the data has to either describe the data in general terms, or be extremely complex. Many solutions for how to disseminate, store and analyze data have been developed. PEDRo [11] is an example of a data schema for how to store and more importantly share proteome data. PEDRo aims to be a comprehensive solution that works for various proteomics technologies. It was developed mainly as a data transfer protocol to aid the information exchange between scientists. PRIDE [12] aims primarily to disseminate and make data publicly available.

Common to most available software solutions is that they are based on database accession numbers (ACs), which of course, are database specific. This is convenient when working with a single experiment search against a single database using one protein identification software package. The usage of database specific ACs breaks down when comparing different experiments searched against different databases. It can also be problematic when searching against the same database at different points in time, since the amino acid sequence belonging to an AC might be updated.

We present an information platform for storage, integration and analysis of proteomic data. Data produced by various technologies can be imported and a number of bioinformatic tools and publicly available databases have been integrated. We have based our software package on primary amino acids sequence to alleviate the problems associated with AC. The number of features and capabilities of the 2DDB software is large and only a selected few will be presented here. This software differs from PRIDE [12] and Peptide Atlas [13] in that the main objective lies in data integration whereas data dissemination has a lower priority.

## Implementation

The software is implemented in Perl 5.8 as an object oriented library, with a CGI script that provides the user interface to the software. The data is primarily stored in a relational database [28], but some data is stored on the file system (mainly to provide input data for third party software, such as BLAST [14]. 2DDB requires a GNU/Linux operating system.

## Results and discussion

The data models are implemented in a relational database [28] and comprise tables ranging from experimental meta-data tables to tables holding results from statistical and bioinformatical analysis. The fundamental unit in the data model is the primary amino acid sequence, referred to as sequence from here on. Each sequence has one or more AC, such as a Swiss-Prot AC [3], associated with it. Basing the database on amino acid sequence alleviates a number of problems. First, the amino acid sequence is database independent in contrast to ACs, meaning that the same amino acid sequence have different ACs in two different databases. Since the sequence is database independent, it is easy to compare experiments searched against different databases. Second, some databases will change the sequence belonging to an AC if the sequence is discovered to be incorrect. It is possible that such a change will corrupt the validity of the identification since the AC was identified by primary sequence. Some databases assigns a single AC to sequences and annotate the polymorphisms or splice variants, whereas others will assign different ACs to each sequence. In our implementation, polymorphisms and splice variants of the same gene will lead to multiple sequence entries. Third, by using primary amino acid sequence is the possible of grouping sequences of high sequence similarity.

In a typical MS experiment, the sequence coverage (the fraction of the full length sequence that was detected in the MS) is low. This is especially true when working with higher eukaryotic systems. This makes it in many cases impossible to discriminate between two sequences differing only by one or a few amino acids. Depending on what software used, different sequences might be selected for the same set of peptides or closely related sets of peptides. This artificially added complexity of the data creates problems when analyzing the data, especially when comparing two independent experiments. We are using a concept we call composite sequence, referred to as MID (multi-sequence identifier), to address the problem. The MID is based on a seed sequence, and sequences similar (95% identical) to the seed sequence are added to the MID still explaining every peptide. The process of adding sequences to MIDs is curated to ensure highest possible accuracy. The MID hence groups together polymorphisms and sequence fragments. The use of MIDs reduces the complexity of the data and makes the subsequent statistical and bioinformatical analyzes more reliable. The MID is not designed to replace the conventional use of ACs, but instead offer a way to convert non-informative ACs to ACs with more information. As the number of sequences increase, so does also the computational cost of keeping the database up to date. Since the MID definition is based on sequence identity and not evalues, it is possible to obtain the data needed to create the MIDs in incremental steps, caching previous results in a lookup table. This table is updated weekly, to ensure full coverage. This way, the computational cost is spread out over time, and is hence also manageable. The first sequence detected is usually the one used as the seed sequence of the MID, but longer sequences are preferred in the case multiple sequences belonging to the same MID is imported at once. When analyzing duplications of experiments, the quality of quantification data is increased by the fact that it is more likely that the experiments share a common MID than an identical identification, especially if a large sequence database was used.

A wide array of analysis tools have been developed for the analysis of the complicated data resource. These tools cover functional, structural, compositional and regulatory aspects of the data. The software package offers a convenient way of importing data from a proteomics experiment, annotate the proteins using one or more of the many tools available and calculate various statistical parameters. It also has the capability to compare experiments.

## The core data model

**Figure 1:**
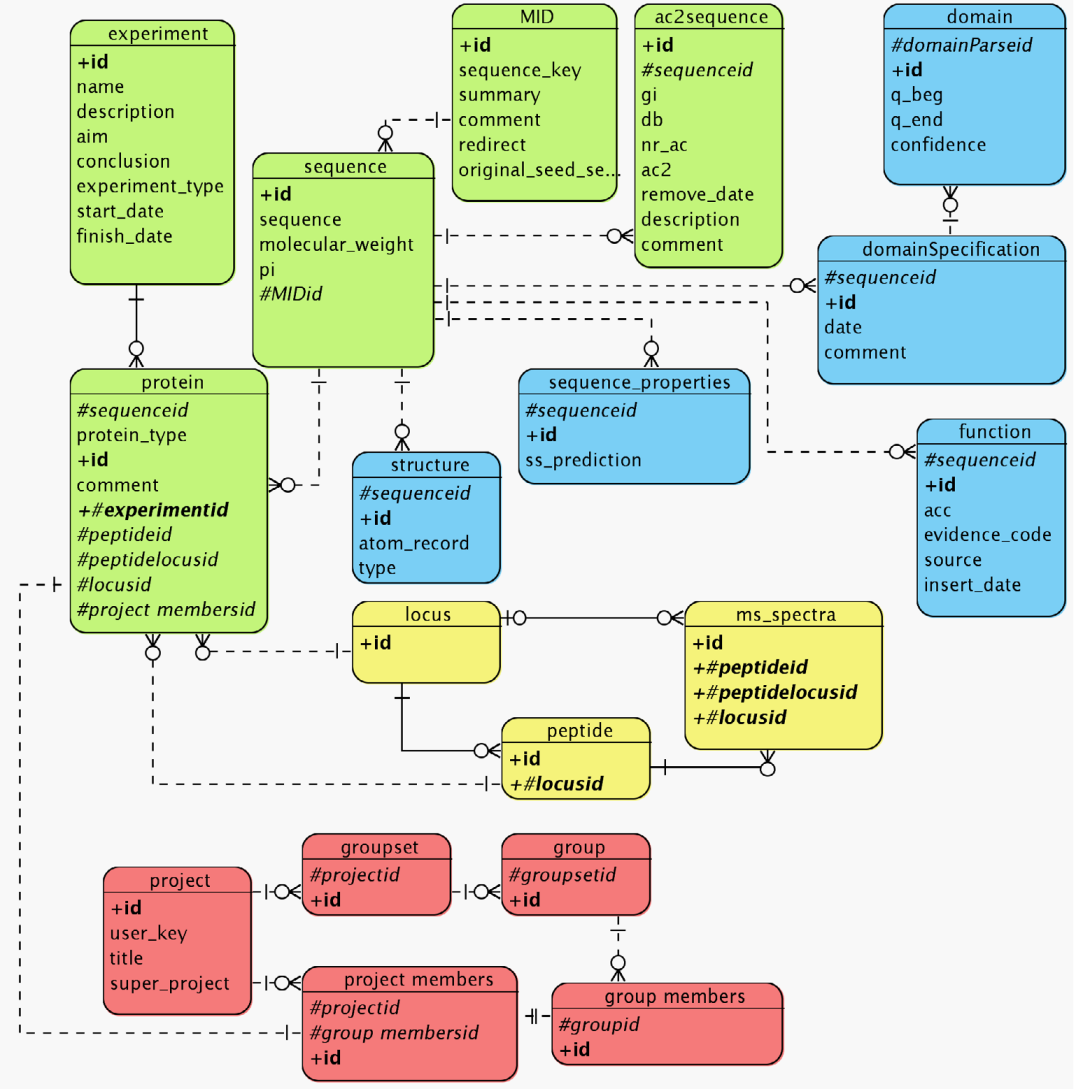
**UML diagram of the core data model**. An UML diagram of 18 of the more important tables of the over 180 tables in the current implementation of 2DDB. The core data model consists of a few tables colored in green. Each box represents a table in the database. The lines connecting the boxes represents relations in the relational database. A relation that ends with a split line represents many entries in the table. A relation with one split end hence represent a one-to-many relation and a relation with two split ends represents a many-to-many relation. One experiment is associated with many proteins (for example the proteins identified in an experiment). Each protein has a single sequence, but that sequence can be present in other experiments hence the one-sequence many protein-relation. Each sequence is associated with one or many accession numbers from different databases, such as Swiss-Prot [3]. In blue are tables that hold information about the sequences. Yellow and red describe the MS related data model and the Protein Explorer respectively. Some tables, such as the peptide table, are extended by several other tables to hold information only relevant to a subset of peptides.

The core data model consists of a few fundamental tables and is the heart of the data model, see Figure 1. Experiments, stored in the experiment table, are loosely defined as a biological sample going through separation, identification and database search. Two different sample preparations naturally end up in two different experiments. Less obvious is that the same spectra search with two different programs or against two different databases end up as different experiments. Each experiment has meta-data associated with it describing the sample preparation, method of separation, protein identification software and so on, and each experiment is associated with one or more proteins. The definition of a protein in 2DDB is the connection between an experiment and a protein sequence and hence a protein identified in two different experiments will have two entries in the protein table. The sequence is stored in a separate table since the same protein can be identified in multiple experiments and it is advantageous to store each unique sequence only once.

## Extended data models

### Mass spectrometry data model

The core data model is not designed to hold experiment specific data and a number of tables had to be added to more accurately handle MS data. This model is capturing the process from separation to identification and handles data from ID and 2D gels as well liquid chromatography-based separation methods (Figure 1). The 2DE module of this software package was published in 2001 [15]. The 2DDB software has since been used in numerous studies, of which a few have been published [16,17]. For example, in one kind of experiment a sample is separated by strong cation exchange and reverse phase chromatography and spotted on a MALDI plate, each spot would be represented as a locus in the database. Each spot is then subjected to MS analysis and the spot is thus linked to one or more MS spectra. Peaks are selected in the MS for fragmentation, and the peptide fragments are subjected to yet another MS analysis and the resulting spectra are assigned a peptide sequence and a protein id by programs such as SEQUEST [1] or MASCOT [2]. Hence each spot is also annotated with one or more MSMS spectra and these spectra are associated with an identified peptide sequence. The peptide sequences are associated with one or more proteins. Note the many-to-many relation between the peptide and the protein table in Figure 1. Similarly, on an LC-MS analysis with an electrospray ionization (ESI)-based instrument, typically a few peptides are eluted of the HPLC at one given time and each data acquisition cycle is initiated with a MS scan followed by a number of MS/MS scans.

Recently, the mzXML [18] standard has gained popularity. It is an open XML format and conversion tools exists to convert mzXML either to or from other popular formats. The benefit with an instrument independent standard is that the instrument specific files can be transformed into the mzXML standard. The 2DDB system is currently supporting this mzXML standard allowing the use of only one set up import tools for all MS-based experiments. Data is easily imported by pointing the system to a file system localization. The system will find XML files, classify them, and imported them. Additionally, if an instrument independent standard is used, only one set of software for further data analysis needs to be developed. Examples of published mzXML based tools are PeptideProphet [10], ProteinProphet [9], ASAP ratio [19] and XPRESS [20], all which is part of the Seattle Proteome Center software pipeline and which are all supported by 2DDB.

A two-dimensional electrophoresis (2DE) separation also fits the data model. In this case a gel spot is a locus. One of the problems with 2DE is that gels have to be matched to one another in order to compare expression of proteins between different samples. This matching is computer intensive and can only be done for a limited number of gels. One of the gels in a matched set is elected to be the master gel, and normally, this is the gel with the most number of gel spots. Corresponding gel spots in every gel in the match set is assigned a unique identifier. 2DDB allows for matching of master gels in different match sets, creating a bridge between match sets. This bridge can then be used to calculate statistics over more gels, which leads to more accurate conclusions. A number of visualization and selection tools make the bridging of the different experiments seamless. Gel slices for bridged spots can be viewed simultaneous to facilitate validation of a match, and ttests can be performed on the fly between groups of different treatments.

### Protein structure and function prediction data model

The functional and structural analyses are implemented in a separate data model. Again, this model is based on the core data model, see Figure 1. The majority of protein bioinformatic tools available depends on the amino acid sequence alone. Thus, all results from the bioinformatic analysis are stored in relation with the sequence table. Each sequence is annotated by various properties. Common annotations include secondary structure prediction [21], trans-membrane domain predictions [22], signal sequence predictions [23] and functional annotations, commonly in the form of gene ontology (GO) terms [24-26]. A large number of tables contain information about these sequences, such as domain information (experimental or predicted), tertiary structure (experimental or predicted), secondary structure (experimental or predicted). Furthermore, various technologies are utilized to find relation between different sequences. Amongst others, we look at sequence similarities, structural similarities and functional similarities. By annotating the sequences with GO terms using sequential and structural information and group the identified proteins on regulation and or co-purification we can assign putative functions to the proteins.

## Protein explorer

**Figure 2:**
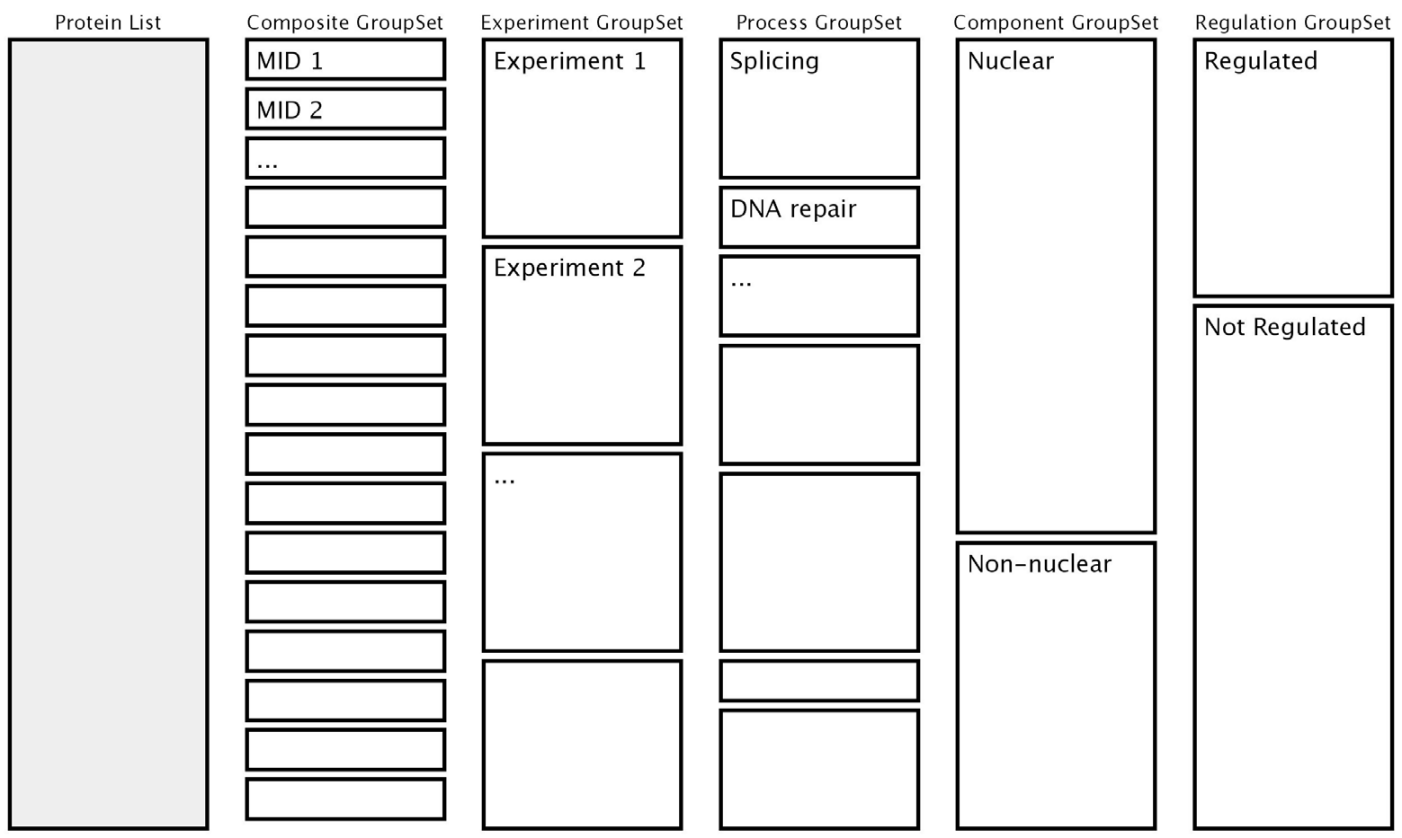
**Protein explorer**. This schematic picture of the protein explorer shows a list of protein, represented as a gray box to the left. This list is called a project. Each protein can belong to multiple groups (only once in each group set). Groups can be generated by automatic scripts such as MID, function, regulation and experiment or by arbitrary user-defined criteria.

Data analysis is difficult when dealing with large data bodies of diverse and complex data. Our solution, called Protein Explorer, is similar to the core data model where we have a few central tables that are then expanded as needed, see Figure 1. Protein Explorer lets the user define a subset of protein from one or more experiments. A set of proteins is called a project. This set of proteins can then be grouped using a number of more or less sophisticated tools, see Figure 2. Groups can be defined on regulation (when expression data is available from for example isotopic labeling or 2DE), size, function, localization and so on. The list of grouping possibilities can potentially be made very long. A set of algorithms to divide the proteins into groups are available and additional algorithms are easy to write and incorporate into the framework. The groups and group sets reduces complexity, allowing the user to focus further analysis of more interesting protein for example induced/repressed proteins from a quantitative data set. Each group has one or more visualization tools in order to display major features of the group. Visualization tools are easily developed and incorporated.

### Normalization and statistics

To normalize data, for example protein expression data, is at times necessary. In 2D gel experiments for example, spot intensities are measured as parts of optical density within the selected group of gels. This works when comparing regulation within the gel set, but fails when comparing between sets and experiments. It is hence necessary to normalize the sets for comparison. Protein Explorer is capable of assigning individual normalization factors to each group in a group set to facilitate complex needs for normalization of expression data. A group of normalization factors are called a normalization set, and more than one normalization set can be defined. This addresses the problem when studying a subset of the data that might be enriched for example all peroxisome proteins seems to be up-regulated when in fact, it is due to an increased number of peroxisomes. Normalizing over just the known peroxisomal proteins will indicate if any of the peroxisomal proteins are specifically enriched.

### Visualization

Each group and group set can be visualized in numerous ways. One can view the forced alignment between sequences in a group, if the group shows co-regulation or not or what functions has been assigned to members of a group. The protein explorer framework simplifies the development of additional visualization and analysis algorithms.

**Figure 3:**
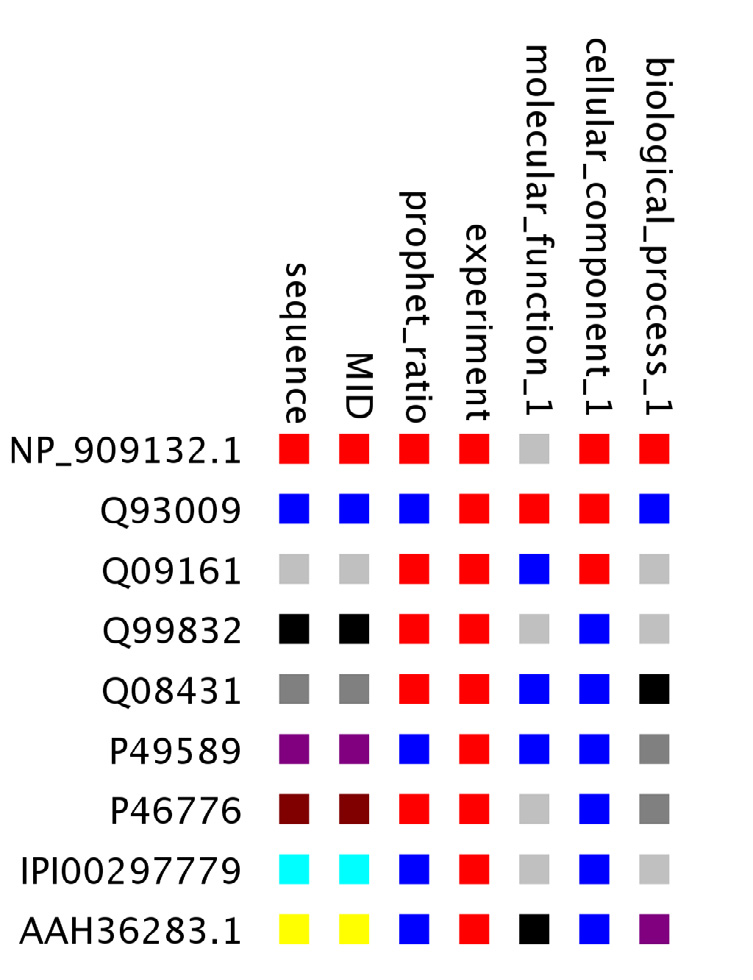
**Color Grid**. The Color-Grid. Proteins are displayed as rows and group sets are displays as columns. The color in each square indicates the identity of the group that the protein belongs to in that group set. The 45 significantly regulated proteins from the experiment discussed in the text is exported into Protein Explorer. Protein Explorer divided the 45 proteins into 7 group sets with between 1 and 45 groups in each group set. The experiment group-set in this case is uninformative as all proteins are from the same experiment. The same is true for both the sequence group set and the MID group set since there are as many groups as there are proteins. The prophet_ratio group set indicates whether the protein was up-regulated or down regulated, and the three go-based group sets are based on MID GO annotations. Only proteins in the cytoplasm (blue group set cellular_component_l) and nucleus (red group set cellular_component_l) are displayed in this image because of limited space).

Group sets can be plotted against them selves or against other group sets in a tool called the ColorGrid. The square in the intersect between two groups can be colored in multiple ways. In the example in Figure 3, all the proteins in a project are listed as rows. To which group it belongs is color-coded along each row. This gives an overview of how similar proteins are divided into groups.

### Application

In an ongoing study, human lung fibroblasts were stimulated with TGF-β_1 _lyzed open and the proteins were reduced, ICAT™ labeled and digested. The peptides were separated by strong cationic exchange and one fraction was analyzed by reversed phase LC-MS/MS by five independent LC/MS measurements and combined. A total of 4731 spectra were assigned to 1825 unique peptides, which in turn identified 1568 proteins all imported into 2DDB. The number of proteins was further reduced to 1518 MIDs by sequence alignment and grouping proteins with a 95% sequence identify still explaining all peptides. This is rather small reduction compared to previous studies [16], the reason being that the ProteinProphet removes the majority of the redundant protein identifications. For example, methionine adenosyltransferas II was reported two times, even though the longer of the two sequences can explain the two unique peptides identified by the mass spectrometer. Both sequences are grouped together into MID M15449.

The second benefit by MID becomes apparent when comparing two of more different experiments. Upon comparing the MIDs present in this study with a previous published study [16] it is possible to immediately see how many proteins were identified in both experiments even though different databases, different search engines and different types of mass spectrometers were used. In this case, when comparing the TCF-β_1 _induced cells with a large scale inventory proteome analysis of the fibroblast nucleus it is possible to draw conclusions of possible sub-cellular localization of the proteins that were in common.

**Figure 4:**
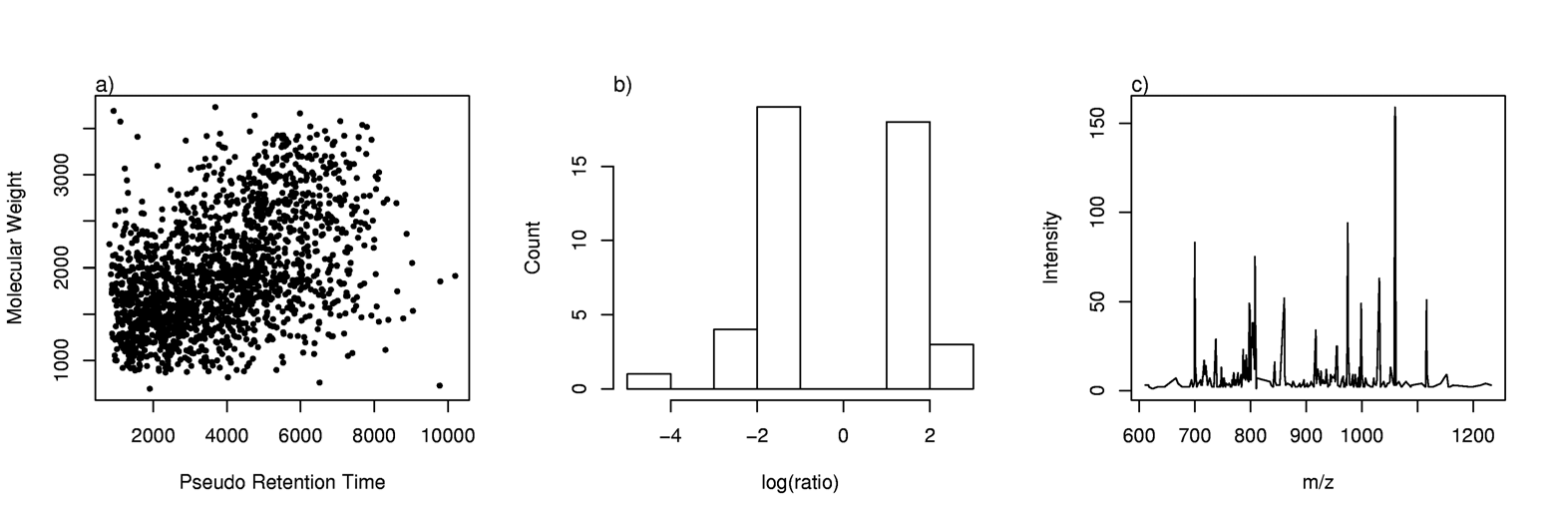
**Visualization of data**. Visualization of data. All these plots are from the experiment discussed in the text. This Figure illustrates four simple graphs designed to give a quick overview of the data, a) is a scatter plot of the theoretical molecular weight of each identified protein over a certain prophet probability (user-settable) against the scan number. The scan number is the sequential number online MS assigns to each spectra it acquires, and since these spectra are acquired with a given time interval, the scan number can be utilized as a pseudo retention time, b) is a histogram of proteins that are significantly regulated (p < 0.05), and a ProteinProphet probability over 0.6. c) is a mass spectra from one of the peptides used to identify HSP72_HUMAN heat shock-related 70 kDA protein 2. These graphs are generated on the fly and enables the user to assess the quality of the dataset.

After the data has been imported and grouped into MIDs several possibilities exits. A number of overview and sorting tools exits which allow the researcher to quickly summarize the data. For example is it possible to obtain an overview of the distribution of database search score (in this case ProteinProphet [10] score) and a histogram of the abundance ratios (Figure 4b). In this case, 45 unique proteins were differently regulated (p-value < 0.05). These 45 proteins, which were imported into Protein Explorer for further analysis, (Figure 3). One can also inspect mass spectra (Figure 4a) and scatter plots of for example retention time and molecular weight (Figure 4c).

Once a smaller set of more interesting proteins is selected, more time consuming analysis can be applied. Thus, the 2DDB software enables the user to import a set of experiments, store the data and to quickly overview the data. By the use of the MID this newly imported set of experiments can be compared to older experiments in the database. A set of proteins can then be specifically exported to the Protein Explorer, in this case 45 differentially expressed proteins, for further in depth analysis. In this particular case, GO have been assigned to the proteins allowing the user to group the proteins based on molecular function, biological process or cellular component to allow functional grouping of the induced/repressed proteins. The GO tool has a graphical component that highlights the found GO assignments in a hierarchical structure. In this way 2DDB facilitates the analysis of complex proteomics data by allowing the researcher to combine, store and overview the data. Typically this is followed by various data reduction steps which enables more in depth analysis of more interesting proteins and in this way functional information of the proteins of interest is provided, which is important when interpreting the results.

Another study involved comparing protein regulation in the cervix between non-pregnant (NP), term-pregnant (TP) and post-partum (PP) women using 2D gels is in its final phase. Three experiment replicates of triplicate gels for each condition was performed. Each experiment was separately matched using PDQuest (BioRad). The master gels from each experiment were matched using the same software and the data from all four match sets were imported into 2DDB. The three first as separate 2DE experiments and the master gel match as a SUPER2DE experiment. PDQuest assigns a unique number, called SSP, to each matched spot and corresponding spots in the three member experiments were linked using the SSPs from the master match set. The software normalizes the individual member experiment allowing to compare them. The statistics becomes more reliable since each treatment has 27 gels instead of 9. One of the significantly regulated spots, Annexin V (Swiss-Prot:P40261), was identified using MS.

## Conclusion

We have developed a software tool that enables fast and accurate analysis of quantitative proteomics data. As more technologies were incorporated it became clear that certain features are universal whereas others are not. The universal features became the core data model and the other features ended up in complementary, technology-specific tables. As a result of this development, the software contains large number of features that are beyond the scope of this article. We have high-lighted some of the more used features most which we think is of interest to the scientific community. We also have tried to display the versatility of the code by illustrating the process of going from raw data to Protein Explorer, checking the quality of the data and enabling conclusions to be drawn. Effort is currently made to extend the display modes in protein explorer.

## Availability and requirements

*Project name*: 2ddb (twoddb)

*Project homepage*:  and 

*Operating system*: LINUX

*Other requirements*: MySQL [28] 4.0 or higher.

*License*: GNU General Public License

*Demo site*: 

## Abbreviations

2DE two-dimensional gel electrophoresis

AC Accession Number

BLAST Basic Local Alignment Search Tool

ESI electrospray ionization

GO Gene Ontology

LC Liquid chromatography

MALDI Matrix Assisted Laser Desorption Ionization

MID Multi-sequence identifier

MS Mass Spectrometry

NR NCBI's non-redundant protein database

## Authors' contributions

LM implemented the software. JM and LM designed the database. JM, TL, GMW and GWT have been involved in the design of the analysis tools, especially protein explorer. They contributed with valuable discussions and feedback during the development. All authors contributed to the writing of this article.
